# Supplementary material for: Tead1a Initiates Transcriptional Priming Through the TEAD1a/YAP‐Notch1‐Spi1/Cebpα Axis to Promote Neutrophil Fate
Source: Adv Sci (Weinh). 2025 Aug 28;12(41):e05441. doi: 10.1002/advs.202505441 (PMC12591127; doi:10.1002/advs.202505441)
Supplement: Supplementary file 4 — Supplementary Dataset [file ADVS-12-e05441-s004.zip › 3-5.zip!/3-5/02-5.cluster/cluster14/Enrich/ego_BP.pdf]

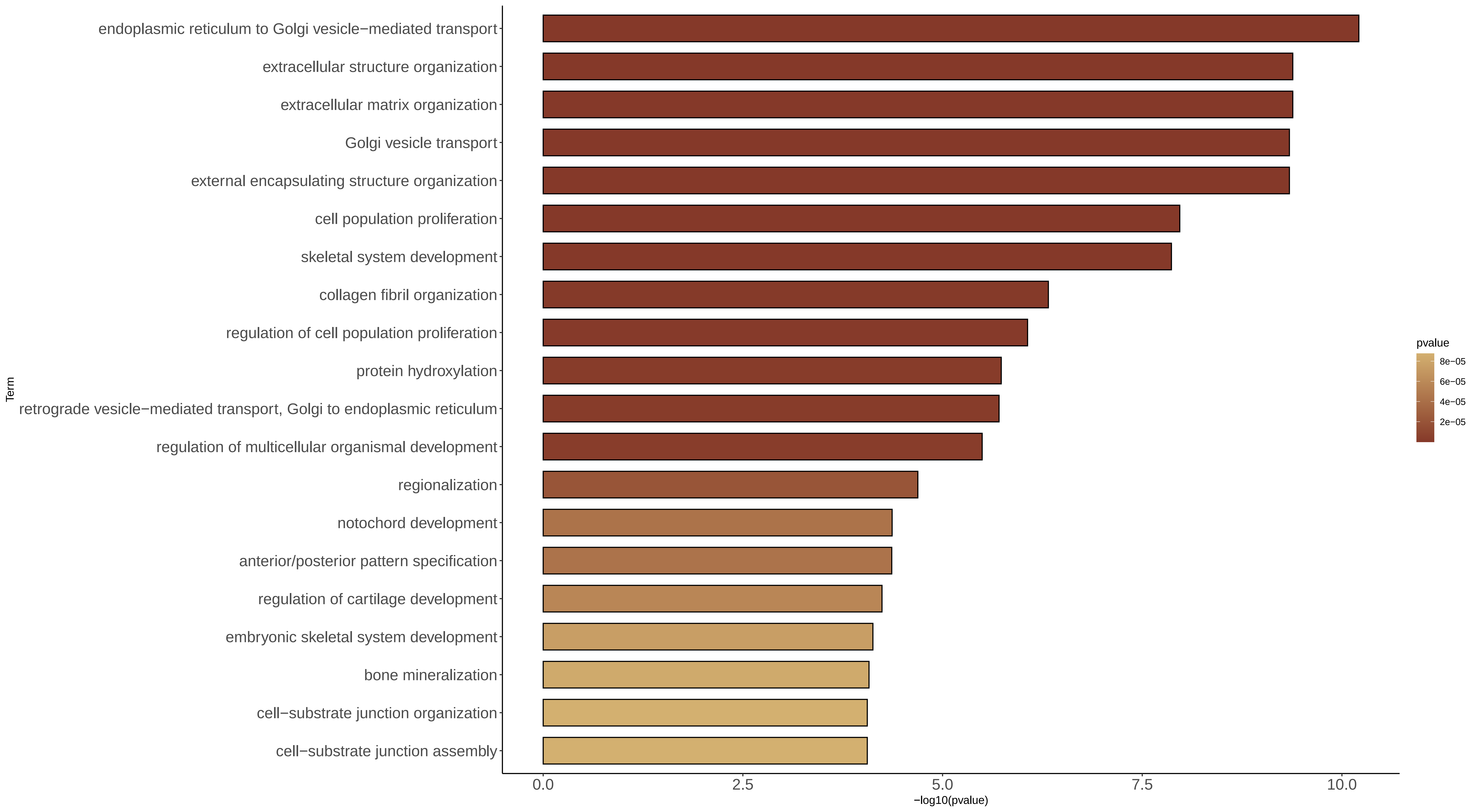

endoplasmic reticulum to Golgi vesicle−mediated transport
extracellular structure organization
extracellular matrix organization
Golgi vesicle transport
external encapsulating structure organization
cell population proliferation
skeletal system development
collagen fibril organization
regulation of cell population proliferation
protein hydroxylation
retrograde vesicle−mediated transport, Golgi to endoplasmic reticulum
regulation of multicellular organismal development
regionalization
notochord development
anterior/posterior pattern specification
regulation of cartilage development
embryonic skeletal system development
bone mineralization
cell−substrate junction organization
cell−substrate junction assembly
Term
0.0
2.5
5.0
7.5
10.0
−log10(pvalue)
pvalue
8e−05
6e−05
4e−05
2e−05
